# Combined enrichment and pharmaceutical intervention reduces excessive arousal in shelter dogs in a randomized controlled trial

**DOI:** 10.3389/fvets.2026.1931203

**Published:** 2026-09-03

**Authors:** Alissa Cisneros, Kristina O’Hanley, Carly M. Moody, Anastasia C. Stellato

**Affiliations:** 1 Department of Animal and Food Sciences, Texas Tech University, Lubbock, TX, United States; 2 Department of Animal Science, University of California, Davis, Davis, CA, United States

**Keywords:** enrichment, excessive arousal, pharmaceutical, shelter dogs, welfare

## Abstract

**Introduction:**

Environmental stressors in animal shelters may lead to development of excessive arousal, posing risks to adoptability and behavioral euthanasia. Recommendations regarding excessive arousal in shelter dogs include providing pharmaceuticals and enrichment.

**Methods:**

A randomized controlled trial was conducted to assess the influence of enrichment and pharmaceutical interventions on shelter dogs (*N* = 50) displaying excessive arousal from a municipal animal shelter in Texas, United States. Data were collected during 1 week of baseline conditions (days 1–7) and 1 week of treatment (days 8–14). Dogs were randomly assigned to a treatment group: (1) baseline (5-min walk, 2x/day), (2) calm enrichment (15-min walks 2x/day,15-min human-animal interaction), (3) active enrichment (15-min play 2x/day, 15-min human-animal interaction), (4) pharmaceutical (20 mg/kg gabapentin, 6 mg/kg trazodone, 5-min walk 2x/day), and (5) pharmaceutical and calm enrichment (20 mg/kg gabapentin, 6 mg/kg trazodone, 15-min walk 2x/day, 15-min human-animal interaction). Arousal scores (0 = no arousal, 4 = extreme arousal) and ease of transition out of kennel scores (0 = no difficulty, 4 = extreme difficulty) were assigned. Behaviors were assessed before, during, and after enrichment provision. All assessments occurred on days 1, 3, 7, 10, and 14.

**Results:**

During post-enrichment, the baseline group had higher arousal scores compared to active enrichment (*p* = 0.041), pharmaceutical (*p* = 0.001), and pharmaceutical and calm enrichment (*p* < 0.001) groups. Arousal scores were lower day 10 (*p* < 0.001) and day 14 (*p* = 0.003; vs. day 1). The pharmaceutical and calm enrichment group exhibited had less lip licking (*p* = 0.004), panting (*p* = 0.032), vocalizations (*p* = 0.003), and jumping (*p* < 0.001) compared to all treatments. The pharmaceutical group (vs. baseline) had less jumping (*p* < 0.001) and more body shaking (*p* = 0.003). Post-enrichment (vs. pre-enrichment) observations had less paw lifting (*p* < 0.001), yawning (*p* = 0.011), vocalizations (*p* < 0.001), and more lip licking (*p* < 0.001), environmental scanning (*p* = 0.002) and panting (*p* = 0.002). Dogs in kennels near exits had less paw lifting (*p* = 0.013) compared to dogs housed in between high arousal dogs. Longer stays were associated with more alert behavior (*p* = 0.001) and less environmental scanning (*p* = 0.04), lip licking (*p* = 0.013), and vocalizing (*p* = 0.029).

**Discussion:**

Results suggest enrichment and pharmaceutical interventions may reduce excessive arousal in shelter dogs and can be used to inform shelter intervention protocols aimed at promoting welfare and adoptability.

## Introduction

Approximately 2.8 million dogs entered shelters or rescues in the United States in 2025, where 54% of dogs had non-live outcomes, such as euthanasia (1). Within shelters, dogs are exposed to a variety of environmental stressors such as noise (2), novelty, unpredictability, confinement for extended periods (3), and minimal human interactions (4). Also, dogs can experience emotional contagion in response to vocalizations from dogs in neighboring kennels (5). Due to prolonged and chronic exposure to these stressors, behavioral changes may be observed, such as the development of excessive arousal (3, 6–9). Excessive arousal describes the level of physiological and behavioral activation in response to stimuli (10). In shelter settings, it is often described as dogs experiencing over-excitement, agitation, stress, decreased behavioral inhibition, and performance of certain behaviors (e.g., jumping, pacing, vocalizing) at high intensity (11–14). Behaviors indicative of excessive arousal have been described as repetitive and are associated with increased activity, restlessness, vocalizations, circling, jumping, and mouthing, observed both inside and outside of the kennel (13). Shelter dogs with excessive arousal may experience welfare impairments, as its performance can indicate negative stress and persistent, unresolved frustration (15, 16). These behaviors may reflect the development of maladaptive coping strategies, as dogs attempt to manage their current state within an environment that offers limited opportunity for appropriate expression. Persistent behaviors may be redirected toward inanimate objects (e.g., destructive chewing of toys, beds) and people (e.g., nipping, mouthing), and may escalate to aggressive-like behaviors (snapping, biting) (13), posing a risk to animal and human safety, increasing risk of euthanasia (17–19). Excessive arousal can also impact the quality of care dogs receive; to maintain control and safety during husbandry tasks (e.g., moving animals), handlers may respond to aroused or difficult-to-handle dogs with more restrictive, forceful handling and fewer positive interactions (20). Further, displays of excessive arousal (e.g., vocalizations, repetitive movement back-and-forth) can also be undesirable to potential adopters, resulting in an increased length of stay in the animal shelter (17–19). If adopted, dogs displaying excessive arousal may be at an increased risk of return, as previous research identified behavioral problems, including hyperactivity, stereotypies, and excessive vocalizations as leading reasons for relinquishment (21). Excessive arousal may also complicate a dog’s adjustment to a new home, as difficulty settling and regulating their behavior can interfere with the development of the human-animal bond, increasing the likelihood of adoption return. Additionally, excessive arousal may impact cognitive performance, as dogs displaying higher level of arousal can have decreased inhibitory control during training (22). Mitigating excessive arousal in animal shelters may therefore support adoption success and adaptation to home environments, either as pet dogs or in working roles (e.g., assistance dogs) (23). These associated risks in dogs displaying excessive arousal in shelters warrant further investigation into intervention strategies. Although excessive arousal can occur during positive and negative experiences (10), the present study focuses on high-arousal, negative-valence states associated with negative stress and difficulty regulating behavioral responses in shelter dogs.

Current recommendations for shelter dogs displaying excessive arousal include increased provision of enrichment (13) and the use of short-term pharmaceutical drugs (24). Benefits of enrichment provision for shelter dogs include preventing boredom, improving handling during husbandry tasks, and reducing the risk of behavior concerns developed due to confinement (25). Enrichment interventions often include increased human-animal interaction and out-of-kennel exercise (13, 26). While research to date has not specifically explored ways to reduce excessive arousal, previous research in shelter dogs has demonstrated reductions in behavioral (e.g., vocalizations, panting) and physiological (e.g., heart rate and cortisol) indicators of negative affect through provisions of positive human-animal interactions, such as petting sessions or physical exercise (4, 27–33). Prior research has also discovered that providing olfactory and auditory enrichment (34), 15-min of vigorous exercise and calm human-animal interactions (35) is associated with reduced performance of arousal-related behaviors (e.g., jumping and vocalizations).

Trazodone, a serotonin receptor antagonist and reuptake inhibitor, and gabapentin, a calcium channel modulator, are common short-term pharmaceutical drugs with anxiolytic effects administered to dogs either separately or in combination (26, 36) to reduce arousal associated with negative affect. Evidence supporting the use of trazodone in dogs in reducing the performance of negatively valanced behaviors (e.g., lip licking, panting, whining) has been observed during veterinary hospitalization (37–39), and for improving adoption success by reducing the length of stay in shelter settings (40). Similarly, the use of gabapentin in dogs has been recommended for reductions in impulsivity, anxiety disorders (36), and in fear-related behaviors (e.g., hiding, body posture, lip licking) (41, 42). Administration of pharmaceuticals has been recommended to help reduce negative affect and arousal levels and increase an animal’s receptivity and responsiveness to behavioral interventions and modifications (25, 26, 43–47). For example, significant reductions in negative affective states were reported in dog owners instructed to pair a personalized behavior modification program with the pharmaceutical imepitoin (46), which has anxiolytic properties similar to gabapentin and trazodone. Also, in shelter cats, significant reductions in stress scores and latency to emerge from hiding were observed in cats administered a behavior modification plan in conjunction with administration of gabapentin (47). Beyond reducing negative states, both enrichment and pharmacological support may also promote positive welfare outcomes (48). These interventions may support adaptive coping within the shelter environment, supporting the development of resilience needed to adjust successfully to a new home. Despite existing evidence on the behavioral effects and relatively affordable nature of these pharmaceutical drugs, no empirical evidence exists to support the use of pharmaceuticals, either by themselves or in combination with behavioral interventions, for reducing excessive arousal in adult shelter dogs.

This randomized controlled study explores the influence of enrichment, including physical activity, human-animal interaction, and pharmaceutical interventions on the behavior of shelter dogs exhibiting excessive arousal, while also examining the influence of kennel location, length of stay, and dog age, sex and weight. Dogs were exposed to one of five treatments (1) baseline, (2) calm enrichment, (3) active enrichment, (4) pharmaceutical, (5) pharmaceutical and calm enrichment, and changes in arousal-related behavior were measured and compared across intervention strategies. We predicted that all treatments would produce reductions in arousal compared to the baseline treatment and that the greatest reductions in arousal would be observed in dogs receiving the combination of enrichment and pharmaceutical strategies, as well as in dogs housed in lower stimulating kennels, such as those located away from high traffic areas.

## Methods

The Texas Tech University Institutional Animal Care and Use Committee reviewed and approved this study (AUP2024-1592).

### Subjects

Dog participants (*N* = 62) were sourced from the Lubbock Animal Shelter & Adoption Center, a municipal shelter in Lubbock, Texas, United States with a housing capacity of 359 dogs. Eligibility requirements included that dogs had completed the 72-h mandatory stray hold period, between the ages of 1 and 8 years old, up-to-date on vaccinations, and had no current medical issues. Additionally, eligible dogs needed to be classified as having ‘excessive arousal’ based on a 5-point arousal scale, developed from prior descriptions of arousal (13). On this scale, excessive arousal is indicated by a score of 3 (i.e., more than three instances of jumping, circling, pacing, and/or vocalizations, with at least a 2-s pause between behaviors), while 0 indicates no arousal (no instances of jumping, circling, pacing, or vocalizations) and 4 indicates extreme arousal (constant jumping, circling, pacing, vocalizations; Table 1). Dogs were assigned separate arousal rankings during both in-kennel and out-of-kennel assessments, each 1 min in length. To assess behavioral eligibility, the investigator (AC) conducted walkthroughs of the kennels to identify dogs displaying arousal-related behaviors (e.g., jumping, vocalizations, pacing, spinning). For each identified dog, the investigator positioned themselves in front of the kennel door and conducted a one-minute behavioral observation to assign an in-kennel arousal score. If dogs scored a 3 (e.g., more than three instances of jumping, circling, pacing, and/or vocalizations, with at least a 2-s pause between behaviors) on the in-kennel portion of the assessment, they were removed from the kennel to complete the out-of-kennel portion of the arousal assessment, which involved a one-minute walk. Dogs that behaviorally presented as a score of 3 for both in- and out-of-kennel assessments were considered eligible for study participation. This threshold was selected to target dogs exhibiting persistent excessive arousal, rather than dogs displaying only moderate or transient arousal responses (score of 2). Requiring dogs to meet the excessive arousal criterion in both contexts was intended to exclude dogs whose arousal may have been limited to anticipation of release from their kennel.

**Table 1:** Five-point arousal scale utilized to score excessive arousal both in- and out-of-kennel during one-minute observations.

Arousal score	In-Kennel description	Out-of-Kennel description
0: No Arousal	No observed jumping, circling, pacing, or vocalizations	No instances of lunging, pulling, jumping, circling, mouthing, or pacing
1: Mild Arousal	Dog exhibits 1–2 instances of jumping, circling, pacing, and/or vocalizations.	Exhibits 1–2 instances of lunging, pulling, jumping, circling, mouthing, and/or pacing
2: Moderate Arousal	Dog exhibits 3 instances of jumping, circling, pacing, and/or vocalizations that are interrupted with at least 2 s between instances.	Exhibits 3 instances of lunging, pulling, jumping, circling, mouthing, and/or pacing and are interrupted with at least 2 s between instances
3: Excessive Arousal	Dog exhibits more than 3 instances of jumping, circling, pacing, and/or vocalizations that are interrupted with at least 2 s between instances.	Exhibits more than 3 instances of lunging, pulling, jumping, circling, mouthing, and/or pacing on walk that are interrupted with at least 2 s between instances
4: Extreme Arousal	Dog exhibits constant (less than 2 s between instances) of either jumping, circling, pacing, and/or vocalizations	Exhibits constant (less than 2 s between instances) of either lunging, pulling with tension on leash, jumping, circling, mouthing, and/or pacing

If any medical issue arose during participation in the study, such as a physical injury (e.g., laceration, lameness), acute illness (e.g., vomiting, respiratory signs), or reduced activity levels, shelter staff were notified and dogs were removed from the study. Additionally, at any point during the study if dogs escalated to a 4 for both the in and out-of-kennel portions of the arousal scale, they were removed due to personnel safety and welfare concerns. As dogs displaying a 4 for either portion of the arousal scale were initially excluded from participation, escalation to this level during the study was considered indicative of behavioral deterioration beyond what the treatment was intended to manage, and continued participation was deemed unethical due to the risk of further distress. Dogs were observed daily by research personnel and shelter staff, and any dog removed from the study due to behavioral concerns was discussed with shelter personnel to facilitate implementation of a more appropriate treatment outside of the scope of the study.

A power analysis (using a 5% margin of error and 95% confidence intervals) was conducted to estimate how many dogs would be needed for this study. To detect differences between the five treatment groups we estimated needing a minimum of 10 dogs per treatment group (total N = 50 dogs); however, to account for potential loss to follow up (e.g., adoption, injuries, illness, behavior concerns), the sample size was inflated to 65 dogs.

### Procedures

**Figure 1:**
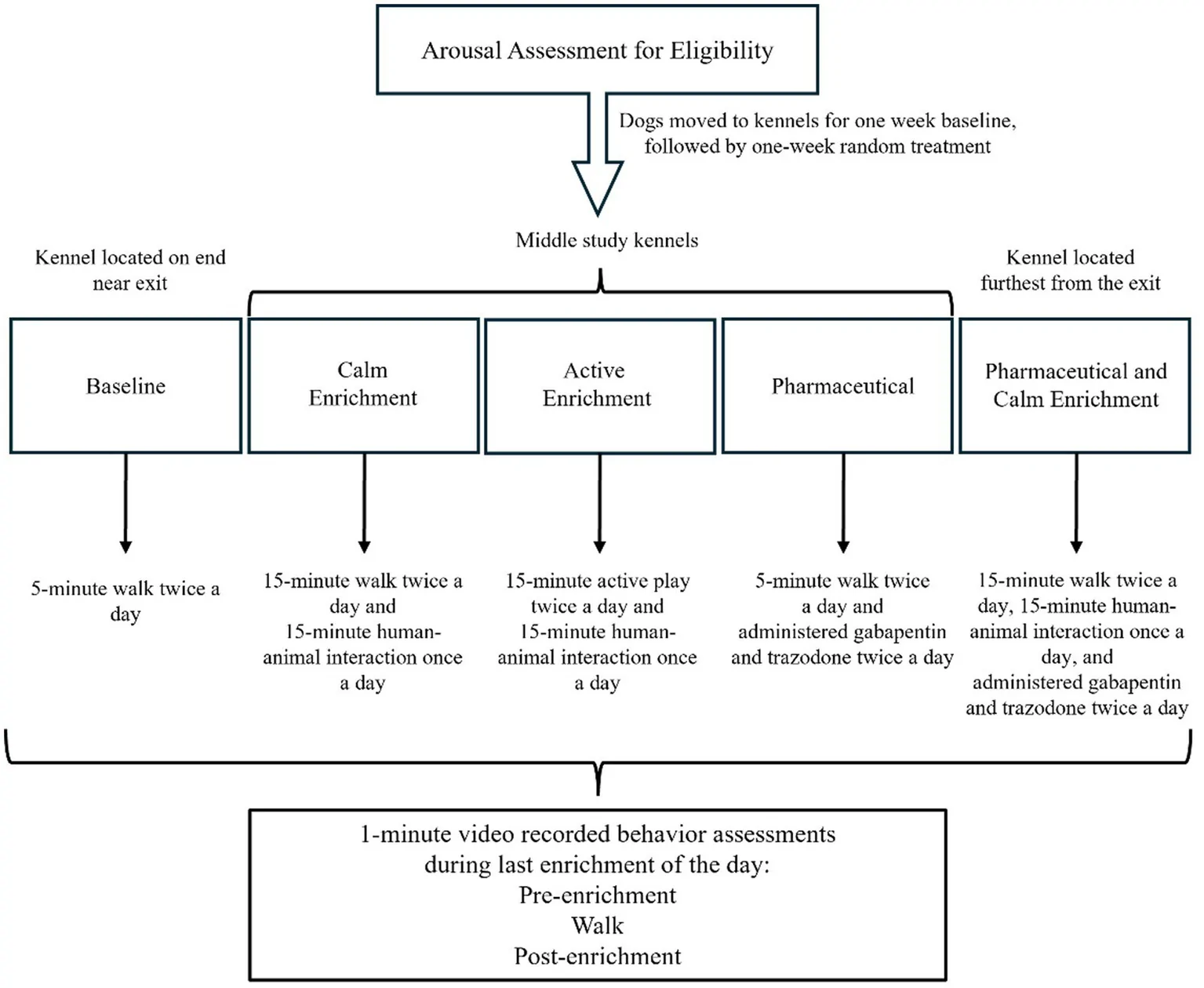
Study procedures including eligibility assessments, followed by movement to one of the five study kennels, that were randomly allocated to a treatment group, and behavioral assessments that were conducted across the control week (Day 1, 3, 7) and intervention week (Day 10 and 14). The kennel location and treatment arrangement shown represents an example following randomization of treatment allocation across the five study kennels.

This study was conducted between March to October 2025. A total of 28 undergraduate and graduate students, all female, from Texas Tech University assisted with data management and enrichment provision (e.g., walks, in-kennel human-animal interaction). Seven of these students were trained to administer pharmaceuticals (i.e., trazadone and gabapentin) and maintain accurate records of medication administration. A summary of procedures can be found in Figure 1.

Dogs at the participating shelter are routinely pair housed and not routinely provided raised beds or toys. Therefore, for the study duration, all study dogs were relocated to five kennels, each facing a wall, that were designated for the study aims (Figure 1). Of the study kennels, one was located at the end of the kennel bank, near the door that exited to the designated walking area or play yard, and they had only one neighboring kennel housing a study dog. Three kennels were located in the middle of the kennel bank, and dogs housed in these kennels had neighboring study dogs displaying arousal on either side. The final kennel was located the furthest away from the exit door and was positioned between two kennels, one with a study dog and the other with a dog from the general shelter population that had varying housing conditions (e.g., single versus pair-housed) and behaviors. Kennel location was randomized and recorded to further assess the differences in housing location on dog behavior. The Association of Shelter Veterinarians’ Guidelines for Standards of Care in Animal Shelters (49) recommend providing shelter dogs with a toy and raised resting area as part of standardized baseline care. Thus, kennels were furnished with a raised bed (40″ x 25” Kuranda Chewproof Elevated Dog Bed, Kuranda Store®) and chew toy (Power Chew Toy Bundle, Nylabone®) and all study dogs were single housed during the experiment. Slip-leads (Fida, Best-Run Technology Co., Ltd., 6 ft. x 1/2″), which are familiar to the dogs housed in this shelter, were utilized during walks. Shelter employees were asked to move five dogs at a time to the designated study kennels at least 48-h prior to study participation to allow acclimation to the modified housing environment. To maintain blinding to treatment allocation, shelter personnel were not instructed regarding which dog should be placed in each kennel. Prior to each group of five dogs being moved, research personnel randomly assigned a treatment to the study kennels using an online random number generator provided by Google Chrome™. As treatment allocation was re-randomized for each cohort of five dogs, this approach ensured that treatment assignment varied across kennel locations throughout the study and prevented treatment effects from being confounded with specific kennel positions (Figure 1).

All dogs received 1 week of baseline conditions that included a 5-min walk twice a day, and 1 week of their assigned treatment. The treatment groups included: (1) baseline, (2) calm enrichment, (3) active enrichment, (4) pharmaceutical, (5) pharmaceutical and calm enrichment. The baseline group continued receiving a 5-min walk twice a day. During walks, research staff used slip leads to walk the dog to the designated walking area and allow them to walk at a moderate pace without running. If the dog stopped to sniff an area, the research staff was instructed to not interrupt this behavior. Treats were provided as needed to redirect the attention of the dog away from other animals. The calm enrichment group received a 15-min walk twice a day and a 15-min in-kennel human-animal interaction once a day. The active enrichment group received a 15-min active play session in a fenced play yard twice a day and a 15-min in-kennel human-animal interaction once a day. The active play session involved placement in play yard with a tug and fetch toy where the dog was encouraged to continuously walk or run by research staff for the duration of the session. The human-animal interaction involved research staff entering the kennel and providing treats, petting, and/or basic training (e.g., sit, lay) while speaking in a calm voice. The interaction continued as long as the dog remained interested; however, if the dog turned or walked away, the research staff was informed to softly call to the dog and provide treats. The pharmaceutical group received a 5-min walk twice a day and were administered a combination of trazodone (approximately 6 mg/kg twice/day) and gabapentin (approximately 20 mg/kg twice/day). The pharmaceutical and calm enrichment group received the same enrichment and pharmaceuticals protocol as the calm enrichment and pharmaceutical groups, respectively. All physical enrichment activities (i.e., walk and play sessions) were conducted between 7 to 9 a.m. and between 4 to 6 p.m., and in-kennel human-animal interaction sessions occurred between 12 to 2 p.m.

Dosages for pharmaceuticals were determined from previous research exploring the impact of trazodone on anxiety and stress during veterinary hospitalizations (38, 50) and the use of gabapentin for behavioral modification in dogs (51). The exact dosages for each dog were calculated based off each dog’s weight which was provided by the participating shelter’s veterinarian. Trazodone tablets and gabapentin capsules were 100 mg each, thus trazodone dosage was rounded to the nearest 50 mg, and gabapentin was rounded to the nearest 100 mg. Pharmaceuticals were administered orally utilizing peanut butter, spray cheese, or wet food on a paper plate twice a day at least 30 min prior to the morning and evening walk. All medication administration was tracked by dosage, time, and research individual administering the medication. All administration of pharmaceuticals was monitored by the shelter veterinarian on staff. Any side effects, such as gastrointestinal upset or severe behavioral suppression, were reported to the veterinarian, who determined whether dosage modifications or removal from the study were warranted. Mild gastrointestinal upset was observed in three dogs receiving pharmaceuticals; however, the signs were short term (1–2 days), and this was not considered severe or abnormal for the shelter population in a manner that would warrant removal from the study or medication cessation based on the shelter veterinarian assessment.

Video recordings occurred on the evening of day 1, 3, and 7 (within the baseline week) and on days 10 and 14 (within the treatment week). Recordings consisted of a one-minute in-kennel observation prior to kennel exit, a one-minute observation during the walk to the designated walk/play area, and a one-minute in-kennel observation immediately upon return to the kennel following completion of the assigned out-of-kennel enrichment protocol. To reduce the potential influence of the presence of research personnel toward the dogs’ behavior, a tripod was set up prior to pre-enrichment and post-enrichment in-kennel observations.

### Data collection

A trained individual blinded to the study hypothesis and treatment assignment assessed video recorded behaviors using Observer XT software (Noldus Information Technology Inc., Wageningen, The Netherlands). Behaviors were scored from three one-minute videos captured during the last physical enrichment session of the day: (1) pre-enrichment in-kennel observation, (2) during the first minute of the outdoor walk, and (3) post-enrichment in-kennel observation. An ethogram of arousal- and fear-related, and general in-kennel behavior was used to guide behavioral observations (Table 2). The ethogram was developed based on prior research on shelter dog in-kennel behavior, including behaviors associated with excessive arousal (e.g., jumping, mouthing, locomotion, vocalizations, alert, environmental scanning) (13, 35), stress (e.g., yawning, panting, reduced posture) (3), resting (e.g., laying) and grooming behaviors) (52), and length of stay (e.g., facing away from the front of the kennel) (52). Continuous sampling was used to score the frequency of discrete behaviors within each one-minute observation video to detect differences in arousal-related behaviors and extent of excessive arousal based on the developed arousal scale. Frequency of discrete behavioral instances, rather than duration, was selected as the primary metric because preliminary video review indicated that dogs either did not display a given behavior or displayed it for the full one-minute observation periods. Alert, facing backwards, grooming, laying, paw lifting, and environmental scanning were only assessed during the in-kennel observations as these behaviors are not distinguishable from expected walking behavior (e.g., paw lifting, environmental scanning) or not applicable during the active out-of-kennel walking assessment (e.g., alert, grooming, laying, facing back of kennel). Vocalizations were live scored as presence or absence during video recordings by research assistants, due to concerns regarding video audio quality due to competing noise levels in the kennel room. To assess observer reliability, observations made by the blinded observer were compared with those made by the investigator (AC), who served as the reference observer. Agreement across all coded behaviors was high (Cohen’s *κ* = 0.91), indicating excellent reliability.

**Table 2:** Ethogram used to assess arousal- and fear- related behaviors as well as body position and general behavior during the observation phase (pre- and post-enrichment, during walk).

Testing phases	Behaviors		Description
All Phases	*Excessive arousal-related*	Jumping	Two or more paws lifted off the ground and may be placed on kennel door, wall, or handler
		Mouthing	Placing mouth around items (e.g., handler’s body, leash, kennel bars) without forceful clamping of jaw
		Vocalizing	Any instance of barking, growling, whining, yelping
		Locomotion	Movement either back and forth or in circular motion, with dog returning to original position
	*Fear-related*	Body shaking	Lateral, side to side rotation of the body about the central axis, with shaking of the fur
		Lip/snout licking	Portion of the tongue moves along the upper lip
		Yawning	Wide opening of mouth
		Reduced body posture	Head low with ears sideways, down or pinned back, and tail lowered or tucked between bent hind legs, either still or wagging
		Panting	Opening of mouth to enhance breathing with tongue visible
		Paw lifting	One paw at a time is raised from ground
In-Kennel	*Body position*	Facing backward	Head and shoulders are oriented toward back of kennel
	*General behavior*	Laying	Underbelly or lateral side of body in contact with the ground
		Grooming	Licking fur and/or use of paws and/or teeth to scratch the body
	*Excessive arousal-related*	Alert	Eyes open with head and ear movement (angular rotations, or moving back-and-forth), with the dog remaining stationary
		Environmental scanning	Eyes moving from one side to the other and back again at least three complete times in succession

Dogs were subjectively scored from video using the same arousal scores (5-point scale ranging from 0 to 4; Table 1) during the eligibility assessments. Additionally, an ease of transition score was developed and modified from the ASPCA Canine-ality™ “Manners and Greetings” scales (53). This scale was used to assess the difficulty in removing dogs from their kennel and brief on-leash walk (~30 s) to the exit door. Behavior (e.g., jumping, locomotion, mouthing) upon the handler approaching the kennel, applying the slip lead, exiting the kennel, and on the brief walk (e.g., assessing extent of pulling on leash) was utilized to assign an ease of transition score. The scale ranges from 0 to 4 (Table 3), where 0 indicates no difficulty in removing the dog from their kennel (e.g., dog did not jump on the kennel door or pull-on leash), and 4 indicates major difficulty (e.g., the dog pushed past the walker in an attempt to escape and constantly pulled on the leash, and exhibited mouthing of the walker, kennel bars, and/or leash). Arousal and ease of transition scores were assigned by one of the investigators (AC) from de-identified videos. Despite de-identification, there was the potential for the investigator to remember treatment assignment; however, the videos were randomized such that they were blinded to assessment day.

**Table 3:** Five-point ease of transition behavior score utilized to determine extent of difficulty to remove dog from kennel and transition to enrichment area (e.g., play yard or walking area).

Ease of transition score	Description
0: No Difficulties	Dog kept all four paws on the ground, was calm and did not jump on kennel or pull-on leash
1: Minor Difficulty	Dog exhibited excitement (e.g., tail wagging, increased activity) but kept all four feet on ground, with pulling on leash
2: Some Difficulty	Dog jumped on kennel door, but not on walker, with pulling on leash
3: Moderate Difficulty	Dog jumped on kennel door and walker with ceasing and pulling on leash
4: Major Difficulty	Dog constantly jumped and pushed on kennel door and walker in attempt to leave kennel, escapes out of kennel past walker, pulled on leash without ceasing, and exhibits mouthing with pressure on walker, kennel, and/or leash

### Statistical analysis

All analyses were performed using Stata Statistical Software© v.17.0 (StataCorp., College Stations, TX, USA). A series of regression models was used to assess the influence of treatment, phase (before, during, after enrichment), assessment day, dog age, sex, weight, and length of stay on arousal- and fear-related and general in-kennel behaviors, and arousal and ease of transition scores. Each model was built using backwards selection, where main effects with a *p* ≤ 0.05 were retained. Dog was included as a random effect to assess the influence of within-dog variation and account for repeated measures. Biologically plausible interactions relevant to the study aims (e.g., treatment and phase) between day, treatment, kennel location, and phase were assessed and retained if significant, and contrast statements were conducted to explore relationships within the detected interactions.

Linear regression models were used to analyze the arousal and ease of transition scores, logistic regression was used to analyze presence versus absence of vocalization and panting, and poisson regression was used to analyze rate of yawning, body shaking, mouthing, and facing the back of the kennel. After assessing anscombe residuals for overdispersion, negative binomial models were utilized when overdispersion was present. Based on findings of overdispersion, jumping, lip licking, locomotion, reduced posture, alert, paw lifting, and environmental scanning was assessed using a negative binomial distribution. Laying and grooming were rarely displayed; therefore, these behaviors were not analyzed. For all models, residuals and outliers were assessed and BIC was used to determine model fit.

## Results

### Descriptives

A total of 50 dogs were included in analysis. Twelve dogs total were excluded due to procedural changes after pilot testing (5), behavioral concerns (i.e., snapping and lunging toward other dogs) observed by shelter employees outside of study procedures (1), medical concerns (e.g., injuries, illness) unrelated to study procedures (3), and adoption (3). Most dogs (56%, 28/50) were 1 year of age (mean age (±SE): 1.82 (0.18) years), with an average weight of 47.2 lbs. The average length of stay was 75 days (range: 15–370 days). All dogs were classified as being mixed breed by the participating shelter.

Pre-enrichment, mean arousal scores for each treatment group, in descending order, were baseline (mean: 3.22, median: 3), active enrichment (mean: 2.88; median: 3), calm enrichment (mean: 2.68, median: 3), pharmaceutical and calm enrichment (mean: 2.10, median: 3), and pharmaceutical (mean: 2.08, median: 2.5). During the walk, arousal scores were the following: baseline (mean: 3.26, median: 3), active enrichment (mean: 3.14, median: 3), pharmaceutical (mean: 3.08, median: 3), calm enrichment (mean: 3.04, median: 3), and pharmaceutical and calm enrichment (mean: 3.00; median: 3). Post-enrichment arousal scores were: baseline (mean: 2.38, median: 3), calm enrichment (mean: 1.98, median: 2), active enrichment (mean: 1.84, median: 1), pharmaceutical (mean: 1.52, median: 1), and pharmaceutical and calm enrichment (mean: 1.2, median: 1).

Ease of transition scores by treatment during the treatment week were the following: baseline (mean: 2.45, median: 2), pharmaceutical (mean: 2.35, median: 2), active enrichment (mean: 2.25, median: 2), calm enrichment (mean: 2.24, median: 2), and pharmaceutical and calm enrichment (mean: 1.95, median: 2).

### Regression results

#### Arousal score

**Figure 2:**
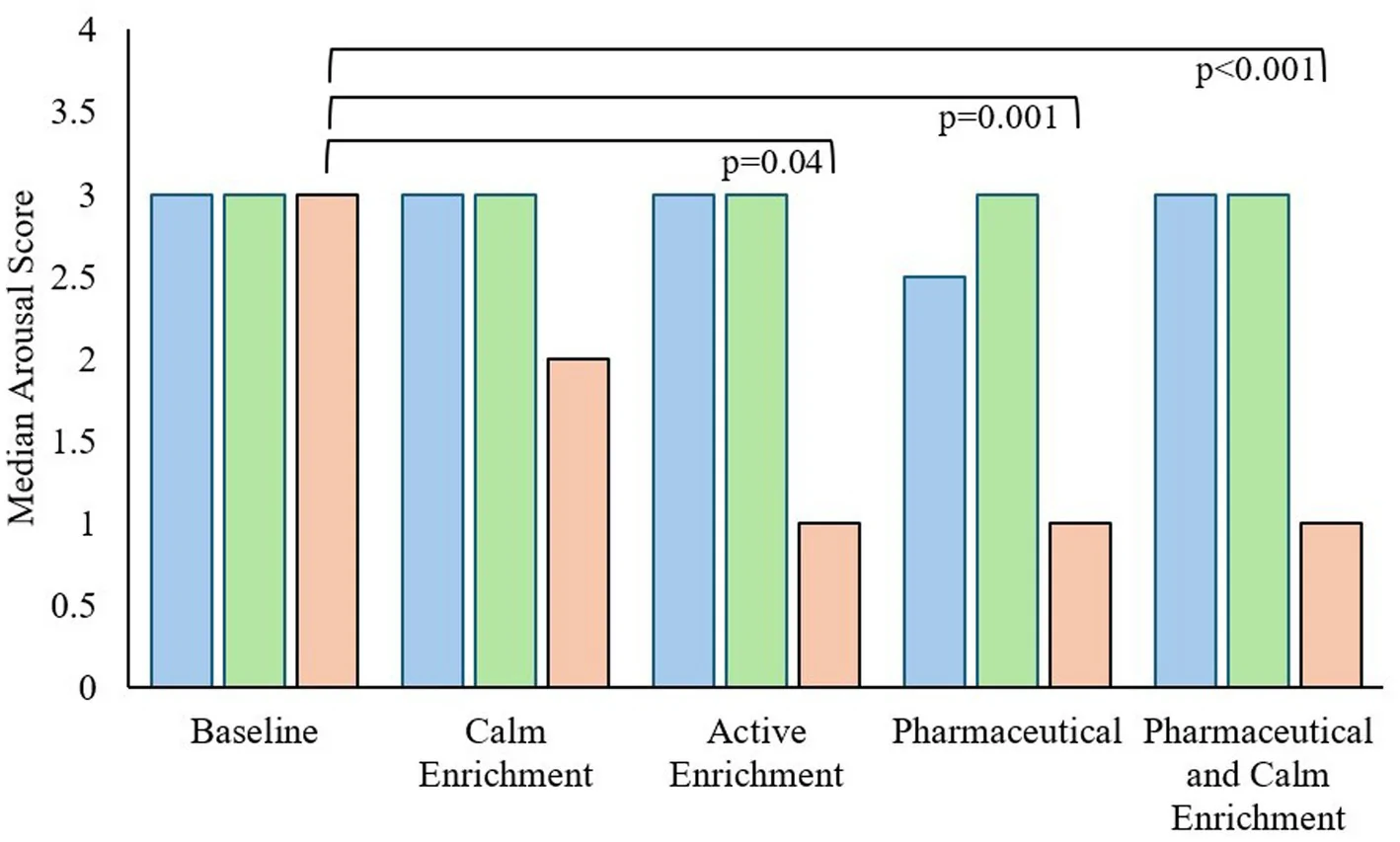
Median arousal score for each treatment by observation phase; pre-enrichment (blue), during walk (green), post-enrichment (orange). During the post-enrichment phase, dogs had significantly higher arousal scores in the baseline group compared to active enrichment, pharmaceutical, and pharmaceutical and calm enrichment groups.

Treatment influenced arousal scores through an interaction with observation phase (*p* = 0.0007), where during post-enrichment, dogs in the baseline group had higher arousal scores compared to the active enrichment (*p* = 0.041), pharmaceutical (*p* = 0.001), and pharmaceutical and calm enrichment (*p* < 0.0001) groups (Table 4; Figure 2). Arousal scores were also associated with assessment day (*p* = 0.0007), with lower arousal scores on day 10 (*p* < 0.001) and day 14 (*p* = 0.003) compared to day 1.

**Table 4:** Mixed linear regression model of assessing factors related to arousal score, with dog as a random effect.

Outcome	Variable		Coefficient^a^	95% CI^b^	*p*-value^c^
Arousal score	Treatment* Observation phase				**0.0001**
		Baseline/Walk vs. Calm Enrichment/Walk	0.22	−0.3, 0.74	0.40
		Baseline/Post-Enrichment vs. Calm Enrichment/Post-Enrichment	0.40	−0.12, 0.92	0.13
		Baseline/Walk vs. Active Enrichment/Walk	0.12	−0.40, 0.64	0.65
		Baseline/Post-Enrichment vs. Active Enrichment/Post-Enrichment	0.54	0.2, 1.06	**0.04**
		Baseline/Walk vs. Pharmaceutical/Walk	0.18	−0.34, 0.70	0.49
		Baseline/Post-Enrichment vs. Pharmaceutical/Post-Enrichment	0.86	0.34, 1.38	**0.001**
		Baseline/Walk vs. Pharmaceutical and Calm Enrichment/Walk	0.26	−0.26, 0.78	0.32
		Baseline/Post-Enrichment vs. Pharmaceutical and Calm Enrichment/Post-Enrichment	1.18	0.66, 1.70	**<0.001**
	Day of assessment				**0.0007**
		Day 3 vs. Day 1	−0.12	−0.31, 0.07	0.23
		Day 7 vs. Day 1	−0.13	−0.32, 0.07	0.20
		Day 10 vs. Day 1	−0.39	−0.58, 0.19	**<0.001**
		Day 14 vs. Day 1	−0.3	−0.49, 0.11	**0.003**

a Coefficient based on output of mixed linear regression where >0 indicates positive relationship and <0 indicates negative relationship.

b 95% confidence interval of the coefficient.

c Bold values indicate statistical significance (*p* < 0.05).

Although kennel location was not significantly associated with arousal score (*p* = 0.64; Table 4), the lowest median arousal score was observed in dogs from the pharmaceutical and calm enrichment group housed the furthest away from the exit door (median = 1), followed by dogs housed between other study dogs (median = 2), and dogs housed closest to the exit (median = 3).

#### Ease of transition score

Treatment was not significantly associated with ease of transition score (*p* = 0.443), nor was observation day (*p* = 0.51), kennel location (*p* = 0.054), age (*p* = 0.918), sex (*p* = 0.108), weight (*p* = 0.132), and length of stay (*p* = 0.83).

#### Behavioral measures

Treatment influenced the display of lip licking (*p* = 0.0097), vocalizations (*p* = 0.038), body shaking (*p* = 0.020), and panting (*p* = 0.032). Dogs from the pharmaceutical and calm enrichment group exhibited lower rates of lip licking (*p* = 0.004) and dogs in the pharmaceutical group exhibited higher rates of body shaking (*p* = 0.003) compared to the baseline group (Table 5). Higher odds of vocalizing was observed for dogs in the calm enrichment group (*p* = 0.003) and higher odds of panting was observed for dogs in the calm enrichment (*p* = 0.003) and active enrichment (*p* = 0.016) groups, compared to dogs in the pharmaceutical and calm enrichment group (Table 6). Treatment also influenced the display of repetitive locomotion (*p* = 0.037) and jumping (*p* = 0.0003) through an interaction with observation phase (Table 5). During post-enrichment, dogs had higher rates of repetitive locomotion (*p* = 0.025) and jumping (*p* < 0.001) in the baseline group compared to the pharmaceutical and calm enrichment group. Dogs in the baseline group also had higher rates of jumping during post-enrichment compared to the pharmaceutical group (*p* < 0.001).

**Table 5:** Mixed Poisson and negative binomial regression models assessing the influence of observation phase, length of stay, kennel, day of assessment, age, and sex on behavior outcomes, with dog as random effect.

Outcome	Variable		IRR^a^	95% CI^b^	*p*-value^c^
Environmental scanning^d^	Observation phase^f^	Post-Enrichment vs. Pre-Enrichment	1.98	1.29, 3.04	**0.002**
	Length of stay		0.99	0.98, 0.99	**0.036**
Paw lifting^d^	Kennel				**0.023**
		Corner Near Exit vs. Middle of Study Kennels	0.12	0.02, 0.63	**0.013**
		Middle of Study Kennels vs. Furthest from Exit	1.55	0.40, 5.95	0.52
	Observation phase^f^	Post-Enrichment vs. Pre-Enrichment	0.09	0.05, 0.16	**<0.001**
Facing back of kennel^e^	Day of assessment				**0.0016**
		Day 3 vs. Day 1	0.71	0.57, 0.88	**0.002**
		Day 7 vs. Day 1	1.10	0.90, 1.33	0.35
		Day 10 vs. Day 1	0.93	0.76, 1.14	0.50
		Day 14 vs. Day 1	1.01	0.82, 1.23	0.96
Alert^d^	Length of stay		1.01	1.004, 1.01	**<0.001**
	Age		1.49	1.17, 1.90	**0.001**
Locomotion^d^	Treatment* Observation phase				**0.037**
		Baseline/Walk vs. Calm Enrichment/Walk	1.45	0.84, 2.52	0.19
		Baseline/Post-Enrichment vs. Calm Enrichment/Post-Enrichment	1.2	0.68, 2.10	0.53
		Baseline/Walk vs. Active Enrichment/Walk	1.52	0.87, 2.64	0.14
		Baseline/Post-Enrichment vs. Active Enrichment/Post-Enrichment	1.27	0.72, 2.24	0.41
		Baseline/Walk vs. Pharmaceutical/Walk	1.38	0.79, 2.40	0.25
		Baseline/Post-Enrichment vs. Pharmaceutical/Post-Enrichment	1.57	0.88, 2.78	0.12
		Baseline/Walk vs. Pharmaceutical and Calm Enrichment/Walk	1.43	0.82, 2.48	0.21
		Baseline/Post-Enrichment vs. Pharmaceutical and Calm Enrichment/Post-Enrichment	1.94	1.08, 3.46	**0.025**
Lip licking^d^	Treatment				**0.0097**
		Calm Enrichment vs. Baseline	0.94	0.72, 1.20	0.61
		Active Enrichment vs. Baseline	1.06	0.83, 1.35	0.64
		Pharmaceutical vs. Baseline	0.93	0.73, 1.19	0.57
		Pharmaceutical and Calm Enrichment vs. Baseline	0.69	0.54, 0.89	**0.004**
	Day of assessment				**0.002**
		Day 3 vs. Day 1	1.11	0.94, 1.32	0.23
		Day 7 vs. Day 1	1.10	0.93, 1.31	0.25
		Day 10 vs. Day 1	0.81	0.68, 0.96	**0.017**
		Day 14 vs. Day 1	0.96	0.80, 1.14	0.61
	Observation phase				**<0.001**
		Walk vs. Pre-Enrichment	0.68	0.59, 0.78	**<0.001**
		Post-Enrichment vs. Pre-Enrichment	1.78	1.56, 2.02	**<0.001**
	Length of stay		0.99	0.997, 0.999	**0.013**
Jumping^d^	Treatment* Observation phase				**0.0003**
		Baseline/Walk vs. Calm Enrichment/Walk	1.31	0.58, 2.93	0.52
		Baseline/Post-Enrichment vs. Calm Enrichment/Post-Enrichment	2.22	0.95, 5.18	0.064
		Baseline/Walk vs. Active Enrichment/Walk	0.77	0.34, 1.72	0.52
		Baseline/Post-Enrichment vs. Active Enrichment/Post-Enrichment	1.57	0.68, 3.64	0.29
		Baseline/Walk vs. Pharmaceutical/Walk	1.2	0.54, 2.68	0.66
		Baseline/Post-Enrichment vs. Pharmaceutical /Post-Enrichment	6.02	2.41, 15.04	**<0.001**
		Baseline/Walk vs. Pharmaceutical and Calm Enrichment/Walk	2.15	0.95, 4.87	0.067
		Baseline/Post-Enrichment vs. Pharmaceutical and Calm Enrichment/Post-Enrichment	12.75	4.70, 34.56	**<0.001**
	Day of assessment				**0.0012**
		Day 3 vs. Day 1	0.82	0.61, 1.11	0.21
		Day 7 vs. Day 1	0.7	0.52, 0.95	**0.024**
		Day 10 vs. Day 1	0.63	0.46, 0.86	**0.003**
		Day 14 vs. Day 1	0.54	0.40, 0.74	**<0.001**
Yawning^e^	Observation phase				**0.0001**
		Walk vs. Pre-Enrichment	0.02	0.003, 0.17	**<0.001**
		Post-Enrichment vs. Pre-Enrichment	0.51	0.31, 0.86	**0.011**
	Sex	Male vs. Female	0.28	0.11, 0.72	**0.008**
Body shaking^e^	Treatment				0.020
		Calm Enrichment vs. Baseline	1.63	0.76, 3.50	0.21
		Active Enrichment vs. Baseline	2.09	0.99, 4.37	0.052
		Pharmaceutical vs. Baseline	2.94	1.44, 5.99	**0.003**
		Pharmaceutical and Calm Enrichment vs. Baseline	0.59	0.57, 2.81	0.56
	Observation phase				**<0.001**
		Walk vs. Pre-Enrichment	27.75	10.23, 75.24	**<0.001**
		Post-Enrichment vs. Pre-Enrichment	2.75	0.88, 8.64	0.083

a Incidence Rate Ratio from mixed poisson and negative binomial models where >1 indicates positive relationship and <1 indicates negative relationship.

b 95% confidence interval of the incidence rate ratio.

c Bold values indicate statistical significance (*p* < 0.05).

d Negative binomial model.

e Poisson regression model.

f Behavior only assessed in-kennel during the pre-enrichment and post-enrichment assessment.

**Table 6:** Mixed logistic regression models assessing influence of treatment, day of assessment, observation phase, weight, and length of stay on behavior outcomes, with dog as random effect.

Outcome	Variable		OR^a^	95% CI^b^	*p*-value^c^
Panting	Treatment				**0.032**
		Baseline vs. Pharmaceutical and Calm Enrichment	2.67	0.65, 10.94	0.17
		Calm Enrichment vs. Pharmaceutical and Calm Enrichment	7.87	1.98, 31.3	**0.003**
		Active Enrichment vs. Pharmaceutical and Calm Enrichment	4.93	1.34, 18.14	**0.016**
		Pharmaceutical vs. Pharmaceutical and Calm Enrichment	1.98	0.55, 7.05	0.29
	Day of assessment				**0.0003**
		Day 3 vs. Day 1	3.40	1.52, 7.61	**0.003**
		Day 7 vs. Day 1	3.06	1.39, 6.75	**0.006**
		Day 10 vs. Day 1	0.84	0.44, 1.63	0.62
		Day 14 vs. Day 1	0.94	0.49, 1.83	0.87
	Observation phase				**0.0035**
		Walk vs. Pre-Enrichment	1.00	0.59, 1.70	1.00
		Post-Enrichment vs. Pre-Enrichment	2.56	1.39, 4.70	**0.002**
	Weight		1.05	1.01, 1.09	**0.013**
Vocalization	Treatment				**0.038**
		Baseline vs. Pharmaceutical and Calm Enrichment	2.78	0.78, 9.98	0.12
		Calm Enrichment vs. Pharmaceutical and Calm Enrichment	6.8	1.88, 24.57	**0.003**
		Active Enrichment vs. Pharmaceutical and Calm Enrichment	3.1	0.86, 10.92	0.084
		Pharmaceutical vs. Pharmaceutical and Calm Enrichment	1.44	0.40, 5.13	0.58
	Observation phase				**<0.001**
		Walk vs. Pre-Enrichment	0.003	0.002, 0.01	**<0.001**
		Post-Enrichment vs. Pre-Enrichment	0.05	0.03, 0.09	**<0.001**
	Length of stay		0.99	0.98, 0.99	**0.029**

a Odds Ratio from mixed logistic regression models where >1 indicates positive relationship and <1 indicates negative relationship.

b 95% confidence interval of the odds ratio.

c Bold values indicate statistical significance (*p* < 0.05).

There were no significant findings for grooming or laying behavior. However, a trend in the data indicates that most occurrences of laying was observed in the pharmaceutical and calm enrichment group (40%, 12/30), followed by the calm enrichment (23%, 7/30), active enrichment (23%, 7/30), and pharmaceutical group (13%, 4/30), with no instances of laying observed in the baseline group. Similarly, for grooming, most instances were observed in the pharmaceutical and calm enrichment (36%, 4/11) and calm enrichment group (36%, 4/11), followed by pharmaceutical (18%, 2/11), and active enrichment (1%, 1/11) group, with no instances of grooming observed in the baseline group.

Assessment day influenced lip licking (*p* = 0.002), facing the back of the kennel (*p* = 0.0015), jumping (*p* = 0.0003), and panting (*p* = 0003; Tables 5, 6). Compared to day 1, dogs were less likely to jump on day 7 (*p* = 0.024), day 10 (*p* = 0.003), and day 14 (*p* < 0.001), face the back of the kennel on day 3 (*p* = 0.002), and lip lick on day 10 (*p* = 0.017), and were more likely to pant on day 3 (*p* = 0.003) and day 7 (*p* = 0.006).

Behavioral responses differed across observation phases for the following variables: scanning the environment (*p* = 0.002), vocalizations (*p* < 0.001), panting (*p* = 0.0035), lip licking (*p* < 0.001), paw lifting (*p* < 0.001), yawning (*p* = 0.0001), and body shaking (*p* = 0.02; Tables 5, 6). Post-enrichment, dogs were more likely to scan the environment (*p* = 0.002), pant (*p* = 0.002), and lip lick (*p* < 0.001), and were less likely to vocalize (*p* < 0.001), paw lift (*p* < 0.001), and yawn (*p* = 0.011), compared to pre-enrichment. During the walk, dogs were more likely to body shake (*p* < 0.001) and less likely to yawn (*p* < 0.001), lip lick (*p* < 0.001), and vocalize (*p* < 0.001), compared to pre-enrichment.

Dog demographic and housing variables were also associated with behavior. Length of stay was associated with alert behavior (*p* < 0.001), environmental scanning (*p* = 0.036), lip licking (*p* = 0.013), and vocalizing (*p* = 0.029; Tables 5, 6). A longer length of stay was associated with increased performance of alert behavior in-kennel (*p* < 0.001), less environmental scanning (*p* = 0.037), lower odds of vocalizing (*p* = 0.029), and lower rates of lip licking (*p* = 0.013). Kennel location was associated with paw lifting (*p* = 0.023), with dogs housed near the exit exhibiting lower rates of paw lifting compared to dogs housed in the middle of the study kennels (*p* = 0.013). Also, dogs with a larger weight had higher odds of panting (*p* = 0.013), dogs older in age had higher rates of displaying alert behavior (*p* = 0.001), and male dogs yawned at lower rates (*p* = 0.008; vs. females).

## Discussion

### Intervention strategies

The current study detected lower arousal scores for dogs provided with active enrichment, pharmaceutical, and combined pharmaceutical and calm enrichment interventions. Dogs provided with calm enrichment did not differ in arousal scores with the baseline group and had increased performance of vocalizations and panting compared to the combined pharmaceutical and calm enrichment group. This suggests that providing additional physical enrichment (i.e., walks) and human-animal interaction (i.e., in-kennel calm interactions including petting, providing treats, and/or basic training) may increase behavioral activation without sufficiently reducing overall arousal. While dogs in the active enrichment group had lower arousal scores compared to the baseline group, differences in individual arousal-related behaviors (e.g., jumping, locomotion) were not observed. The provision of pharmaceuticals alone resulted in lower arousal scores compared to the baseline group, and this effect was accompanied by some corresponding changes in behavioral indicators of arousal, including lower rates of jumping and more body shaking compared to baseline group. Further exploration into pharmaceutical intervention, including the impact of trazodone or gabapentin alone compared to a combined approach could aid in developing informed recommendations. Dogs that received a combination of pharmaceutical and calm enrichment exhibited lower arousal scores and lower rates of vocalizations, lip licking and panting compared to other treatment groups and showed differences in arousal-related behaviors across observation phases, including reduced jumping and repetitive locomotion after receiving enrichment. While dogs receiving the active enrichment group had lower arousal scores compared to the baseline group, differences in individual arousal-related behaviors (e.g., jumping, locomotion) were not observed. Taken together, the data suggests that the combination of enrichment and pharmaceuticals produced the most consistent behavioral changes indicative of reduced excessive arousal compared to other treatments. These findings support the use of a combined intervention approach and aligns with previous research and recommendations on the benefits of using pharmaceuticals to support behavioral modification (25, 26, 43–47). Future research exploring a combined active enrichment and pharmaceutical approach could further strengthen recommendations on the effectiveness of type of physical enrichment used in combination with pharmaceutical interventions.

Three treatment groups, calm enrichment, active enrichment, and the combination of pharmaceutical and calm enrichment, had the same 15-min in-kennel human-animal interaction session, yet arousal outcomes differed markedly across these groups. Dogs receiving calm enrichment showed no reduction in arousal scores, while dogs receiving active enrichment and the combination of pharmaceuticals and calm enrichment showed reduced arousal scores compared to baseline. This pattern suggests that the additional intervention (e.g., physical activity or pharmaceutical) paired with the human-animal interaction was the more influential driver of reduced arousal. Active play between humans and dogs may act as a modulator of excessive arousal, as heterospecific play in other species, such as rats, has been shown to reduce fear-related behavior (54). Further research is needed to explore this role of play in shelter dogs. Although prior research has indicated reductions in vocalizations and panting in shelter dogs following 30 min of positive human-animal interaction (e.g., petting interactions) (30), and a single 15-min petting session has been associated with relaxation-related behaviors (e.g., reduced standing, soliciting contact) (31), these studies did not assess the influence of previous arousal-related behavior or temperament on outcomes. Results from this study suggest that these interactions alone may not be sufficient for shelter dogs displaying excessive arousal without pairing them with an additional intervention. In the present study, duration of human-animal interaction was held constant across treatment groups and interaction type was unstructured. As research assistants were instructed to either pet, talk to, provide treats, and/or train the dogs, further research is needed to explore the impact of varying interaction types and duration on behavioral displays of excessive arousal in shelter dogs.

Regarding the influence of assessment day, results suggest that regardless of treatment, providing a structured enrichment schedule can support a reduction in arousal, as there were lower arousal scores and less lip licking and jumping during the intervention week compared to day 1 of the baseline week. Additionally, within the baseline week, there was some evidence of behavioral activation and arousal, with increased panting and a lower likelihood of facing the back of the kennel compared to day 1. These findings may reflect acclimation to the study environment and/or anticipation of enrichment activities as dogs became accustomed to the study routine and associated opportunities for social and physical engagement. Thus, providing routine enrichment schedules for dogs exhibiting excessive arousal has the potential to impact in-kennel behavior for shelter dogs displaying excessive arousal.

### Kennel location

Kennel location only influenced the performance of paw lifting, a behavior often associated with stress (55) with dogs housed in kennels near the exit exhibiting less paw lifting compared to dogs housed in the middle between other dogs displaying excessive arousal. Though it was hypothesized that increased exposure to conspecifics with excessive arousal would have contributed to increased arousal scores through the process of emotional contagion, where the emotional states and behaviors (e.g., vocalizations, jumping) of nearby dogs influence the behavioral responses of surrounding animals (5), there was no difference in arousal scores across kennel locations. It is likely that being in a room with approximately 200 dogs contributed to a high level of auditory and visual environmental stimulation, thereby minimizing any location-specific effects on arousal. Additionally, dogs housed near the exit door, despite having only one neighboring study dog, may have been exposed to more stimulation as other dogs needed to be walked past their kennel to enter or leave the room. These factors may have offset any reductions in arousal further contributing to the lack of observed differences between kennel locations. Given the potential for emotional contagion and conspecific exposure to contribute to excessive arousal in shelter environments, shelters often house dogs displaying excessive arousal away from high-traffic areas; however, the effectiveness of this practice has not been empirically evaluated. Further research conducted in more controlled settings is needed to determine whether housing dogs adjacent to conspecifics displaying excessive arousal, as well as kennel location characteristics such as proximity to exits and high-traffic areas, influence the expression of arousal-related behaviors.

### Other factors

Behavioral differences were also observed across observation phases. During the walk there was less lip licking, yawning, and vocalizing, and more body shaking; whereas after enrichment, there was less paw lifting, and more environmental scanning, lip licking, and panting. These results suggest that during the walk dogs exhibited a temporary reduction in in-kennel behaviors typically reported to be associated with stress, frustration, and in-kennel excessive arousal (11–14) as they transition to enhanced activity outside of the kennel. In contrast, after the enrichment, dogs seem to be more behaviorally activated and engaged with their surroundings, as further evidenced by dogs in the baseline group displaying increased locomotion after enrichment provision. These findings align with prior literature exploring the influence of enrichment on in-kennel dog behavior, which reported increased movement upon return to the kennel after 15-min of active play (35). Further research is needed on long-term impacts of physical exercise on a dog’s ability to settle upon return to their kennel.

Length of stay was associated with displays of vigilance-related behaviors (e.g., alert, environmental scanning). Dogs with a longer length of stay were associated with less environmental scanning, lip licking and vocalizations, and more alert behavior. These results may suggest that dogs exhibiting excessive arousal may alter how they monitor their environment from active (i.e., scanning the environment) to passive vigilance (i.e., alert but remaining stationary) over time. It remains unclear whether this pattern reflects adaptation to the shelter environment, habituation to repeated stimuli, or the development of alternative coping responses during prolonged shelter housing. Further research is needed to explore the impact of length of stay on the development of arousal-related behavior in shelter dogs.

### Limitations

This study was conducted within a single municipal shelter with a housing capacity of 359 dogs at one time, where environmental stressors such as elevated noise, human activity, and conspecific exposure were common. While this setting allowed for the evaluation of dogs within a highly stimulating shelter environment where excessive arousal was frequently observed, findings may not directly generalize to lower-volume shelters with different housing conditions and environmental stressors.

All dogs enrolled in the study were relocated to different kennels furnished with a raised bed and chew toy to meet basic recommended standards of kennel provisions (43), were provided the recommended minimum of two daily walks (56), and many transitioned from pair- to single-housing. Adjusting their environment in this way may have impacted expression of behaviors independent of treatment. For instance, behaviors such as facing the back of the kennel and jumping were reduced prior to treatment provision; however, significant changes in arousal scores were not observed until the intervention week assessments. Though results support the potential value of simply improving housing conditions with the provision of basic resources and time out of kennel, these changes alone are unlikely to fully account for treatment-related differences and therefore additional interventions are still needed to meaningfully reduce excessive arousal in shelter dogs. Recommendations to increase provision of enrichment and administer pharmaceuticals, including the baseline level of care of two walks a day, may exceed what many shelters are able to provide. As such generalizability of these findings may be limited by variability in shelter staffing and resources. While this study aimed to identify feasible interventions for dog displaying excessive arousal, individual shelter variation may still impact intervention success. Future research is warranted to explore additional enrichment strategies suited to shelters with more limited time or resources to allocate toward these interventions. Further, despite behavioral differences observed across the study period, the intervention period was only 1 week; therefore, the long-term effectiveness of these interventions remains unknown. Longitudinal research is needed to evaluate whether pharmaceutical and enrichment-based interventions produce sustained reductions in excessive arousal over time. Additionally, research is needed to explore the impacts of discontinuation interventions on arousal levels in shelter dogs. This work is particularly important for pharmaceutical interventions, that are not intended for long-term use and may be reduced or discontinued following behavioral reassessment.

Pharmaceuticals were administered using peanut butter, spray cheese, or wet food to ensure reliable consumption. While necessary for administration, the foods provided constitute a form of nutritional enrichment, thus some of the behavioral improvements observed in the pharmaceutical and pharmaceutical and calm enrichment groups may partly be attributable to the palatability and consistency of this food provision rather than the pharmacological effects alone. Though treats were also provided during human-animal interaction and walk sessions, the quantity and consistency of treat delivery varied, with no fixed schedule. Future research could address this potential confound by administering an empty capsule or unmedicated food items to non-pharmaceutical groups.

Observed behaviors may have also been influenced by factors outside of the study’s control, such as shelter staff handling during daily husbandry tasks (e.g., cleaning) and dog behavioral history and prior experiences. The source population in the participating shelter is composed of generally younger and larger, mixed-breed dogs. Despite attempts to balance age and size through a randomized block design, the available demographic limits generalizability of results to different age groups and breeds. Further research may be beneficial in exploring age and breed effects on the performance of excessive arousal as well as how these factors may influence the efficacy of interventions.

Assignment of treatment was randomized to kennel location for each cohort of dogs to reduce the potential for investigator or shelter staff bias. Despite shelter staff being blind to treatment allocation by kennel, shelter staff still retained the responsibility of selecting which dog was moved into each of the five study kennels. As a result, staff perception of the dog’s temperament, dog demographics (e.g., size, breed, age), and prior experience with the dog could have influenced kennel placement. For example, shelter staff may have placed dogs that they perceive as more difficult to handle in the kennel closest to the exit to reduce the time needed to walk them outdoors. Further research examining staff perceptions and handling practices in relation to dogs displaying arousal-related behavior could help inform recommendations surrounding kennel placement for dogs displaying excessive arousal.

Another limitation of the current study is that dogs were enrolled based on the presence of excessive arousal-related behaviors without knowing the underlying motivation contributing to development of those behaviors. Excessive arousal may arise from a variety of factors, including anxiety, frustration, motivation to engage with humans and conspecifics, and general high activity levels. Consequently, dogs displaying similar behavioral presentations may differ substantially in their underlying behavioral needs and therefore their responsiveness to specific interventions. Future research should therefore incorporate these behavioral assessments and investigate whether tailoring interventions according to underlying causes improves treatment outcomes.

## Conclusion

This study explored various commonly recommended interventions for reducing excessive arousal in shelter dogs. Providing pharmaceuticals alone, active enrichment, and pharmaceuticals combined with calm enrichment reduced arousal scores, though the most consistent reductions in arousal scores and individual arousal-related behaviors resulted from providing pharmaceuticals combined with calm enrichment (extended walks and positive human-animal interactions). Future research is needed to explore long-term impacts of these interventions and to develop individualized interventions while remaining feasible for implementation in shelter environments.

## Data Availability

The raw data supporting the conclusions of this article will be made available by the authors, without undue reservation.
